# Year-round distribution, activity patterns and habitat use of a poorly studied pelagic seabird, the fluttering shearwater *Puffinus gavia*

**DOI:** 10.1371/journal.pone.0219986

**Published:** 2019-08-06

**Authors:** Martin Berg, Jannie F. Linnebjerg, Graeme Taylor, Stefanie M. H. Ismar-Rebitz, Mike Bell, Chris P. Gaskin, Susanne Åkesson, Matt J. Rayner

**Affiliations:** 1 Centre for Animal Movement Research, Department of Biology, Lund University, Lund, Sweden; 2 Department of Bioscience, Aarhus University, Roskilde, Denmark; 3 New Zealand Department of Conservation, Wellington, New Zealand; 4 Experimental Ecology - Benthos Ecology, GEOMAR Helmholtz Center for Ocean Research Kiel, Kiel, Germany; 5 School of Biological Sciences, University of Auckland, Auckland, New Zealand; 6 Wildlife Management International Limited, Wellington, New Zealand; 7 Northern New Zealand Seabird Trust, Warkworth, New Zealand; 8 Auckland Museum, Auckland, New Zealand; CEFE, FRANCE

## Abstract

We present the first study to examine the year-round distribution, activity patterns, and habitat use of one of New Zealand’s most common seabirds, the fluttering shearwater (*Puffinus gavia*). Seven adults from Burgess Island, in the Hauraki Gulf, and one individual from Long Island, in the Marlborough Sounds, were successfully tracked with combined light-saltwater immersion loggers for one to three years. Our tracking data confirms that fluttering shearwaters employ different overwintering dispersal strategies, where three out of eight individuals, for at least one of the three years when they were being tracked, crossed the Tasman Sea to forage over coastal waters along eastern Tasmania and southeastern Australia. Resident birds stayed confined to waters of northern and central New Zealand year-round. Although birds frequently foraged over pelagic shelf waters, the majority of tracking locations were found over shallow waters close to the coast. All birds foraged predominantly in daylight and frequently visited the colony at night throughout the year. We found no significant inter-seasonal differences in the activity patterns, or between migratory and resident individuals. Although further studies of inter-colony variation in different age groups will be necessary, this study presents novel insights into year-round distribution, activity patterns and habitat use of the fluttering shearwater, which provide valuable baseline information for conservation as well as for further ecological studies.

## Background

Procellariform seabirds are known to undertake long migrations to exploit seasonally favourable feeding areas far from their breeding grounds [1, 2, 3]. These migrations are often crucial for recovering body condition and for replacement of flight and body feathers prior to the next breeding season [4]. Foraging success in non-breeding areas may, consequently, have carry-over effects on breeding phenology and reproductive success in migratory birds [5, 6, 7]. For instance, Bogdanova and colleagues [8] linked breeding success or breeding failure of black-legged kittiwakes (*Rissa tridactyla*) with contrasting geographical distributions in the non-breeding season. Studying the year-round distribution and foraging behaviour of seabirds is, for this reason, fundamental for understanding how events at sea may impact individual fitness and population dynamics [9, 10]. Understanding more about the ecological links of seabird spatial behaviour is further of particular importance given that the rapid decline of many seabird populations can result from factors such as reduced prey abundance, changed oceanographic conditions and fisheries interactions at sea, far away from the breeding grounds [11, 12, 13].

Although developments of miniaturised tracking technology have provided new insights into the foraging strategies and at-sea distribution of many seabird species [2, 14, 15, 16, 17], some seemingly common species remain poorly studied. This is partly because the introduction of light-weight instrumentation only recently has allowed tracking of smaller species, such as *Puffinus*-shearwaters [15]. One example is the fluttering shearwater (*Puffinus gavia*), a medium-sized (~330 g) burrow nesting Procellariform endemic to northern and central New Zealand [18, 19]. Because of its small size and limited distribution, no detailed study of the foraging ecology has been carried out on this species prior to this study.

Fluttering shearwaters are abundant in New Zealand coastal waters year-round with large flocks, sometimes numbering thousands of birds, and a common sight in Auckland’s Hauraki Gulf the austral winter months [19]. The species is known to forage over shallow coastal waters, making them vulnerable to coastal fisheries and water pollution from terrestrial sources, which is why understanding the behaviour and at-sea distribution of this species is crucial for an effective conservation management. The diet of fluttering shearwaters has not yet been studied, but flocks are often seen foraging on “bait balls” of schooling forage fish and Euphausiids that are forced to surface waters by marine mammals and large predatory fish (Authors’ pers. obs.). Total population size of fluttering shearwaters has been estimated as 100,000+ breeding pairs [20]. However, the actual population size may be much lower than this estimate and requires further study [19, 21]. At-sea sightings along the coast of southeastern Australia indicate that fluttering shearwaters migrate over the Tasman Sea during the non-breeding season. However, in the absence of tracking data, these migrations have remained poorly understood.

In this study, we used miniaturised geolocation-immersion loggers (also termed Geolocators, Global Location Sensors or GLS-loggers) in combination with bathymetry data to examine the seasonal movements and activity patterns of fluttering shearwaters breeding on Burgess Island and Long Island in northern and central New Zealand, respectively. This new information is not only relevant in the context of conservation management, and especially in relation to gillnet fisheries, but will likely also motivate further ecological studies of several aspects of the life history of this poorly studied seabird species.

## Methods

### Ethics

The attachment of GLS tags to fluttering shearwaters was covered under a Department of Conservation Wildlife Act Authority number: 38016-FAU and Animal Ethics Committee approval No. 218 issued by the Animal Ethics Committee for tracking of oceanic seabirds project, issued October 2010. Fieldwork on Long Island was conducted on Department of Conservation land and all permissions for work on Long Island were from the Department of Conservation and their Animal Ethics committee.

### Fieldwork and logger attachments

**Fig 1:**
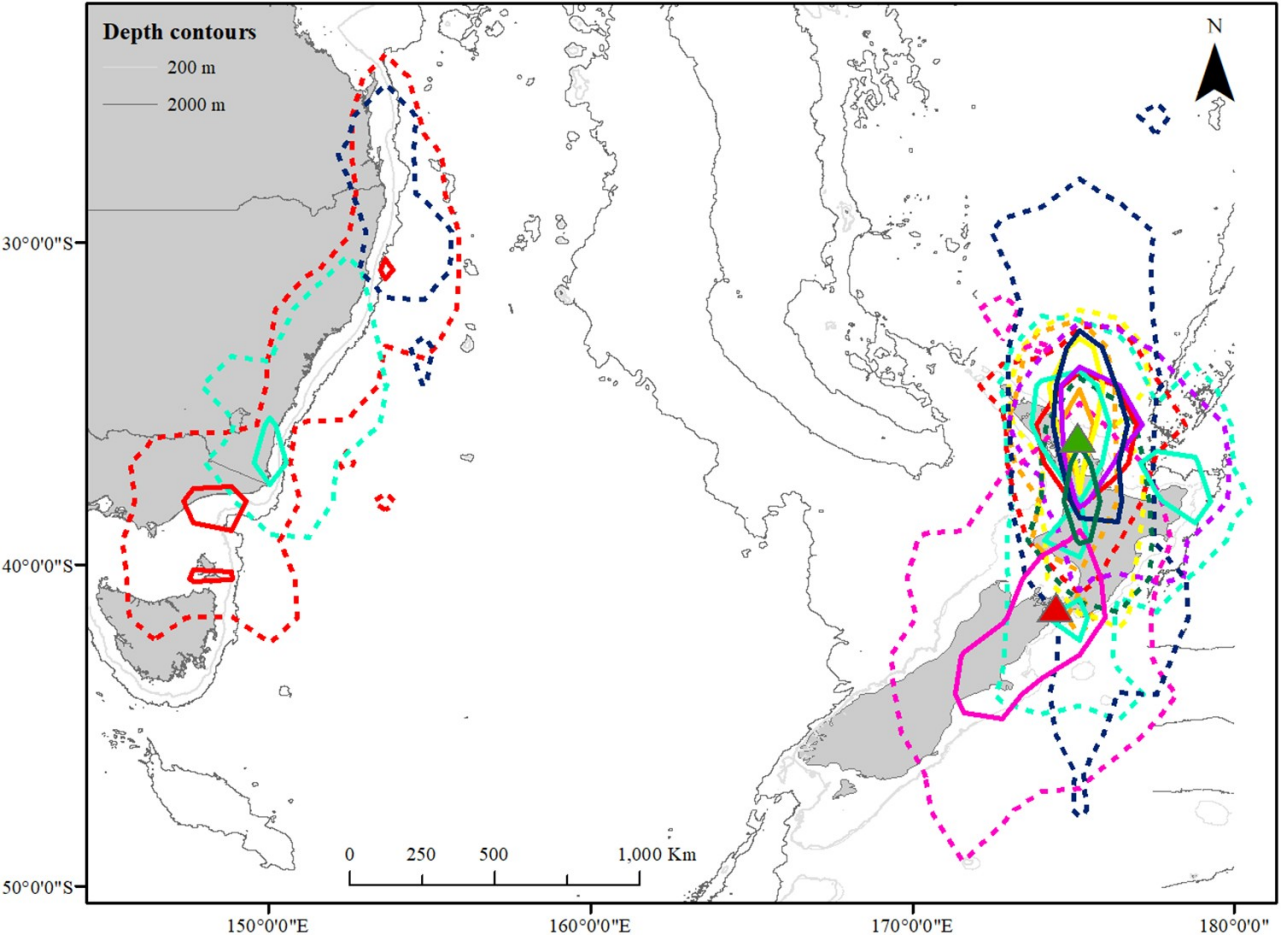
50% (solid line) and 95% (dashed line) kernel distribution of eight individually coloured fluttering shearwaters tracked with geolocators from Burgess Island (n = 7; green triangle) and Long Island (n = 1; red triangle) throughout one to three annual cycles (2011–2014). The individual breeding on Long Island is represented by pink lines. Depth contours at 200 m and 1000 m to visualise shelf slopes and seamounts. The map was created in ArcGIS 10.5 and the bathymetry layers produced according to metadata as in Whiteway, 2009 [59], and Mitchell et al. 2012 [60].

Loggers were attached and retrieved at breeding colonies on Burgess Island (35°54'S, 175°06'E) and on Long Island (41°07'S, 174°06'E; Fig 1) in New Zealand during the early incubation period in the austral spring in September 2011, 2012, 2013 and 2015. Incubating adult fluttering shearwaters were captured in their burrows by hand and placed in cotton bags for weighing and further handling. Geolocation-immersion loggers model Mk15 (2.4 g) and Mk18 (2.0 g) (hereafter referred to as “loggers”) produced by the British Antarctic Survey, Cambridge, were attached to 16 birds on Burgess Island and 1 bird on Long Island. Loggers were attached to a Darvic leg band using two small ultraviolet-resistant cable ties following established protocols [22]. The combined deployment weighed <1% of the body mass of the smallest study bird (298 g) and was thus well under the threshold of 3%, above which deleterious effects are more likely to occur [23]. The loggers were retrieved across three years (2012–2015) on Burgess Island, and in 2013 on Long Island. Darvic leg bands were removed at recapture, and the birds’ legs were examined for any signs of damage. Birds were sexed using a universal method for molecular sexing of non-ratite birds [24].

### Analysis of movement patterns

Light-level data was analysed in BASTrack software to provide approximate positions twice daily corresponding to local noon and midnight with a mean error and standard deviation of the mean of around 186 ± 114 km [25]. A standard light threshold of 10 light units was used to identify day/night transitions. Dark periods less than 6 hours were filtered out to reduce the effects of burrow attendance and ambient light. Loggers were ground-truthed at the colonies and the data was analysed with a sun elevation angle of -3.5°. The sun elevation angle (angle below horizon) was set by ground truthing and comparing derived latitudes with known visits to the colony by each bird.

We inspected the integrity of each light curve with the TransEdit application (British Antarctic Survey). Longitude was derived from the time of local midday with respect to Greenwich Mean Time, and latitude was derived from day length. To filter unrealistic position estimates, we removed positions which; (1) obtained light curves showing interference at dawn and dusk, (2) were within 20 days on either side of spring and autumnal equinoxes, and (3) were separated from sequential locations requiring an unrealistically high flight speeds (i.e. > 2000 km in 24 hrs) [15]. To reduce the influence of outliers caused by the average positional error, all locations were smoothed using a three-position moving average based on spherical trigonometry, i.e. after converting to 3D Cartesian (x,y,z) coordinates [26]. In accordance with previous studies [15], patterns of core and general at-sea distribution were examined for each individual with 50% and 95% kernel density contours. Kernel densities were calculated and analysed by applying the *kernelUD* function in the *adehabitatHR* package in R3.5.3 [27, 28] and incorporated into the Geographic Information System (ArcGIS 10.4). We used least square cross validation (LSCV) to estimate the smoothing parameter. The core foraging area within the kernel density output was calculated in ArcGIS 10.4.

While inland trips are extremely unlikely, locations inland have not been removed since this procedure would have a biasing effect on overall distribution centres [15]. We also estimated four phenological and spatial parameters for every complete migration cycle: (1) Arrival date, (2) duration of time spent in breeding areas, (3) departure date, and (4) duration of time spent in non-breeding areas. Departure and arrival times were considered to be the last and first day when the bird’s location inside the breeding or non-breeding 50% kernel calculated individually for each bird. Distance travelled from breeding colonies was calculated with the Haversine Formula [29] by calculating the great-circle distance in kilometres between Burgess Island and Long Island respectively, and the daily positions of each bird. Analysis of bathymetry was done with the R-package *Marmap* [30], where each foraging location was plotted against bathymetric and topographic values in meters.

### Analysis of activity patterns

Activity data were recorded every 3 sec and provided an immersion value (from here denoted ε) of 0 (100% dry) to 200 (100% wet) corresponding to the sum of positive readings during 10 min intervals. We identified three types of behaviour from the salt-water immersion data: (1) time flying or at the colony: the sum of all 10-minute intervals with ε ≤ 3 (mostly dry). The upper level of this threshold is set to account for occasional splashes where the bird only briefly touches the water [22]. (2) Sitting on the sea surface: the sum of all 10-minute intervals with ε ≥ 195 (mostly wet). The lower threshold is set to account for incidental behaviours, including stretching and scratching, which can trigger intermittent dry readings when the bird, in fact, was resting on the water surface. (3) Probable foraging: the sum of all 10 minute blocks with 3 < ε < 195 (intermediate). Prey is taken by pursuit diving into the water with partly folded wings or is seized from the surface while moving forward on the water surface with the head under water and wings raised (Authors’ pers. obs.). Seizing prey from the surface may also be recorded as intermittent wet-dry, as the activity repeatedly involves splashing and short flight bouts (Authors’ pers. obs.). Intermittent wet-dry 10-minute readings likely also include non-foraging behaviours such as preening, intense scratching, stretching, and conspecific interaction, but at least reflect periods of active movements and increased energy expenditure [31, 32]. Since immersion data was assessed over 10 min intervals, we removed probable foraging (from now referred to as “foraging”) readings that occurred during only one 10 min period between floating and flying/at the colony (or vice versa). These episodic readings probably indicated that the bird went from flying to landing (or vice versa) during the transitional period and was not foraging. Note that most other studies apply the number of “flight bouts” defined as the number of isolated dry intervals as a proxy for foraging activity instead of the threshold method applied in this study [33, 34, 35]. Here we apply the threshold method because the combination of our activity data and our observations at sea indicate that fluttering shearwaters, in contrast to many other Procellariiformes such as albatrosses and gadfly petrels, do not adopt a “fly-and-forage” strategy, and the number of feeding events is therefore not clearly correlated with the number of flight bouts.

The proportion of each behaviour was grouped into “day” and “night”, where the day was defined as the start of civil twilight in the morning to end of civil twilight in the evening (sun < 6° below the horizon [36]. To compare monthly differences in foraging behaviour we compared the number of minutes per day spent floating, flying/at the colony, and foraging per day and night in proportion to the total number of daily daylight and darkness 10 minutes blocks. To examine daily differences over a 24-hour period we grouped each behavioural category into the total number of minutes per hour in proportion to the total number of days during the investigated period, and applied the local sunrise and sunset to define day and night. We also identified days spent in the burrow by the occurrence of complete daytime darkness in the logger traces, while >4h sustained night-time dryness allowed for the identification of burrow visits during the night.

### Statistical analysis

All data was analysed in R3.2.1 [28] involving the packages dplyr, Rmisc, lattice, and ggplot2 [37, 38, 39, 40]. Distributional normality was tested by plotting the data, applying a qqline, as well as applying Shapiro-Wilk’s normality tests. We used paired t-tests to compare the overall differences in time spent foraging, resting, and flying between daylight hours and darkness. We also used a One-way ANOVA to test if all tracked fluttering shearwaters spent more time foraging during daylight hours or in darkness. A student´s t-test was used to compare location bathymetry between migratory and resident birds and daily flight distance between migratory and resident birds. A paired t-test was also used to compare birds with and without loggers to make sure the devices had no negative effect on the weight of the birds. All results are presented as the standard deviation of mean (S.D.). P-values <0.05 were considered significant.

## Results

### Data retrieval and impacts

Out of 17 deployments in 2011, 2012, 2013 and 2015, 13 adult birds with loggers (76%) were retrieved on Burgess Island, and one logger (100%) on Long Island from one to three seasons after devices were attached. The single logger deployed on Long Island was recovered in September 2012. Of these 14 loggers, six loggers failed to download data resulting in eight complete datasets from two females, four males and two birds where the sex remained unknown. One bird was tracked for three consecutive years, one bird for two consecutive years, and six birds were tracked for one year each. A total of 6099 data locations were obtained, of which an average of 19 ± 8% was excluded, as they did not generate realistic positions (see Material and methods), leaving 4969 locations for analyses. Birds with loggers did not differ significantly in mass prior to and following logger deployments (t_6_ = 0.44, p = 0.67), indicating that the devices did not have a negative impact on the birds.

### At-sea distribution and migration

**Fig 2:**
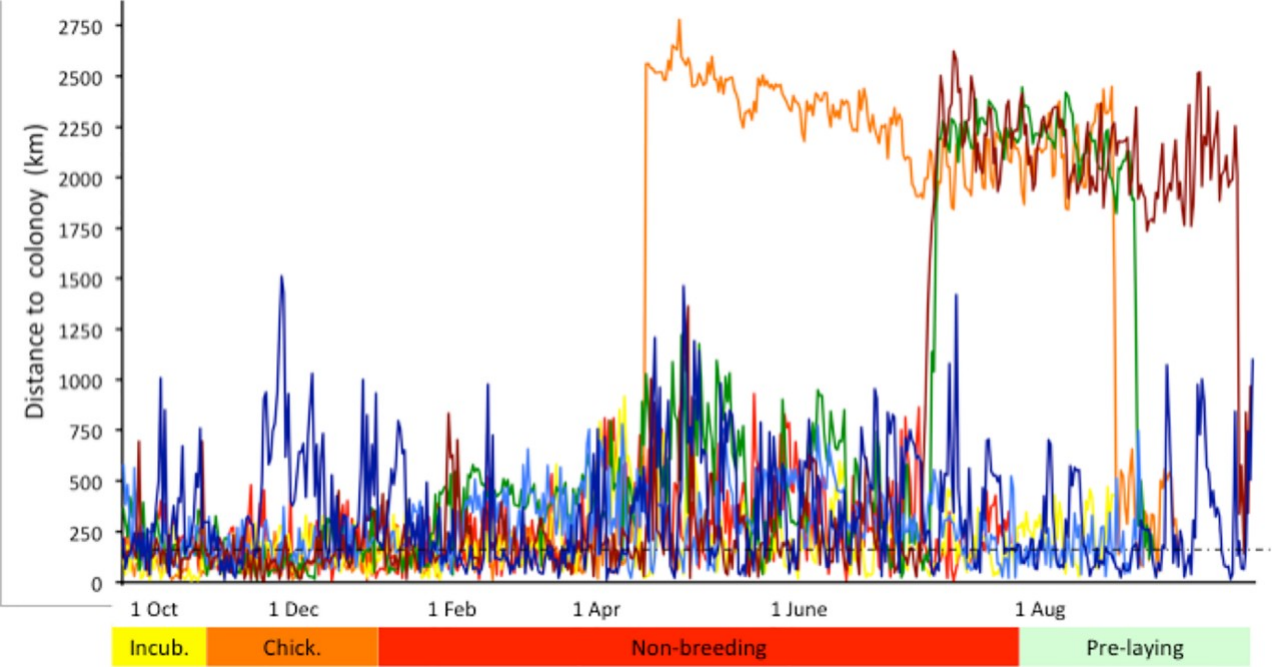
Great circle distance of eight adult geolocator-tracked fluttering shearwaters from their breeding colonies during the year 2011–2015. Incub. = incubation, chick. = Chick-rearing. Dashed line at 186 km indicates accuracy of geolocators.

**Fig 3:**
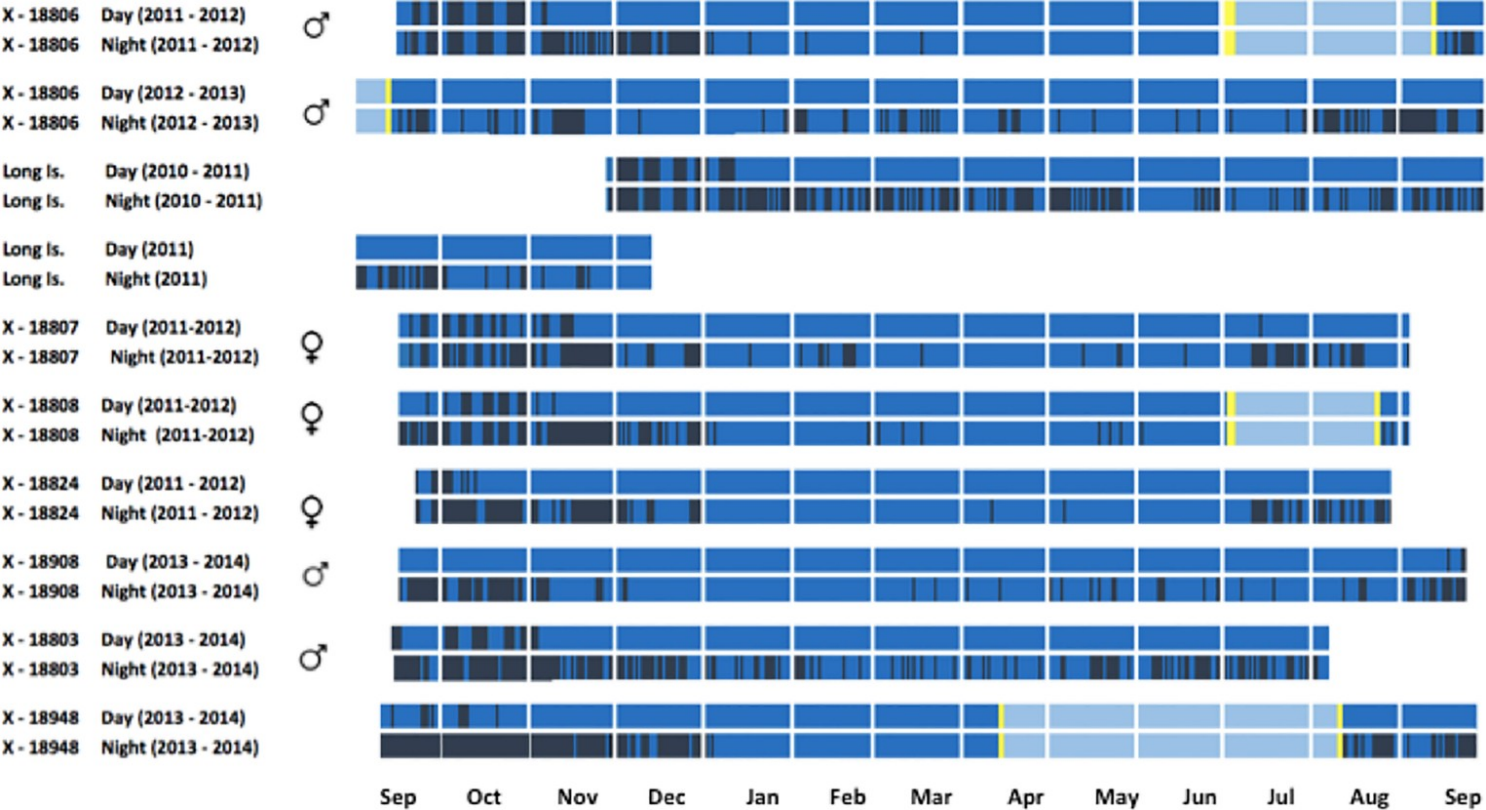
Individual phenology of daytime and nighttime traces represented for eight fluttering shearwaters *Puffinus gavia* breeding on Burgess Island and Long Island labelled with ring number. The largest proportion of each trace (dark blue) represents time spent foraging around New Zealand. Pale blue represents time spent foraging along the coast of E Australia. Active migration over the Tasmanian Sea is represented by yellow. Dark bars represent time spent on land at the breeding colony (>4 hrs/day or night).

We generated kernel density maps for seven birds tracked from Burgess Island and one bird tracked from Long Island over 2790 days (median: 350 days, range 350–657 days) (Fig 1). Five (two females, two males, and one bird of unknown sex) out of eight tracked birds stayed close to their breeding colony year-round (average distance to colony 276 ± 225 km) (Fig 2). All tracked individuals frequently visited their colony at night throughout the year (Fig 3). The Hauraki Gulf was within the 50% core range year-round for birds breeding on Burgess Island and the Marlborough Sounds was within 50% core range year-round for the bird breeding on Long Island. The fifty percent core range increased almost four-fold during the non-breeding season for resident birds increasing from 98,908 km^2^ during chick rearing in November-December to 369,968 km^2^ in April-May and 386,304 km^2^ in July-August.

Of the seven birds breeding on Burgess Island, three birds (one female, one male and one bird of unknown sex) conducted a non-breeding migration to the coast of eastern Tasmania and Australia, while four individuals remained close to the breeding areas year-round, indicating two different winter migration strategies. The bird breeding on Long Island remained confined to New Zealand waters year-round.

Migratory individuals showed a lack of synchrony on departure from breeding grounds leaving on 14 April, 1 July and 2 July, and they returned on 12 August, 14 September and 22 August, respectively (Figs 2 and 3). The bird that arrived in Australia in mid-April foraged in coastal waters around eastern Tasmania and Bass Strait before shifting its distribution northwards along coastal waters of New South Wales before reaching southern Queensland in late July to early August where it stayed confined to waters around Fraser Island until its departure back to New Zealand. The two other birds arriving in Australia in July were foraging almost entirely in coastal waters along northern New South Wales and southern Queensland close to Fraser Island. The bird that migrated back to New Zealand in mid-September did not breed this following season. These birds spent 115, 86 and 46 days along the Australian coast, while the migration over the Tasman Sea was rapid and with no stopovers, lasting 2–3 days (Figs 2 and 3).

We found that resident birds travelled on average 289 km/day and migratory birds as much as 492 km/day, the latter being influenced by the crossing of the Tasman Sea. Because of the inherent error associated with light-logging (186 ± 114 km [25]), we suggest future studies to use GPS loggers in order to get more reliable data.

### Habitat utilization

**Fig 4:**
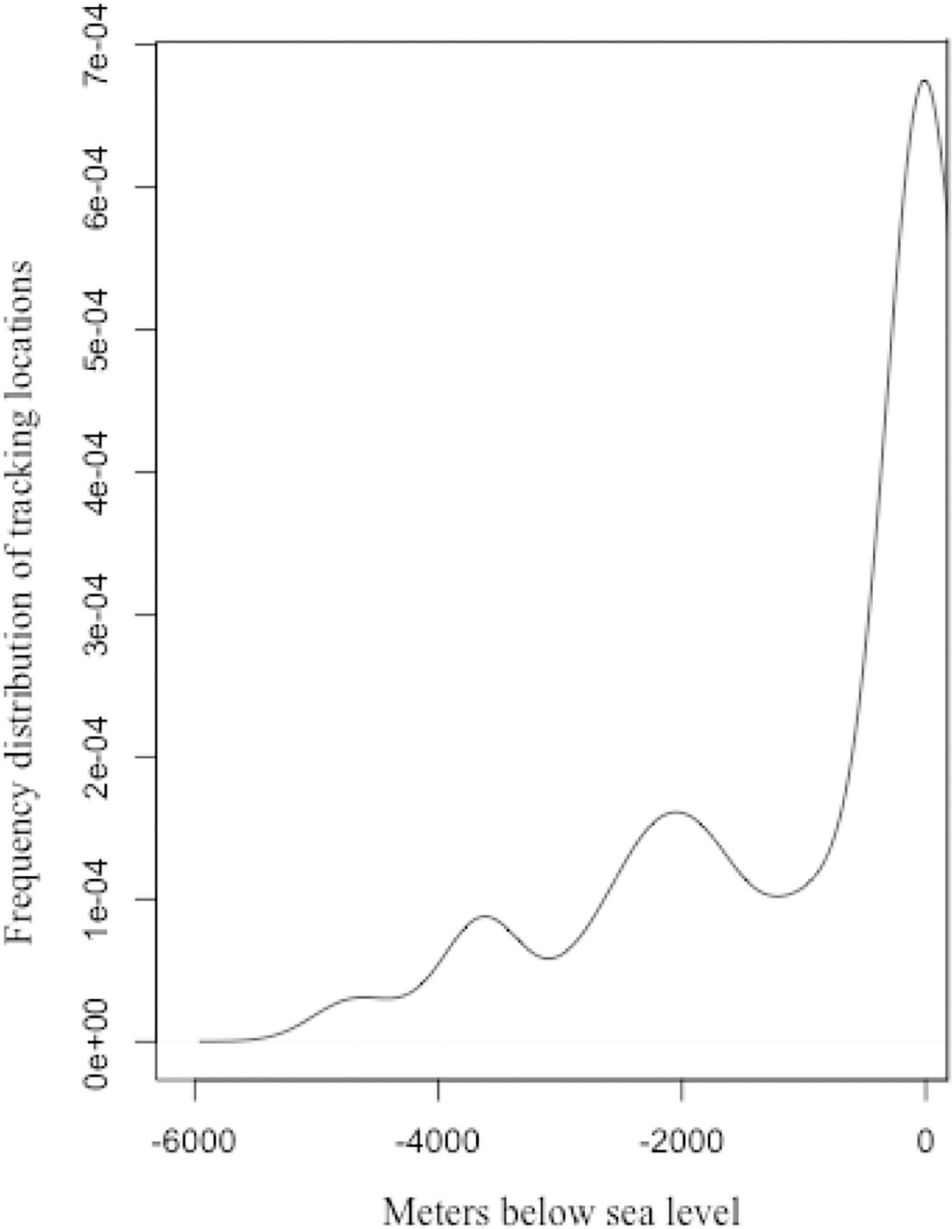
Year-round frequency distribution of the tracked fluttering shearwater locations in relation to oceanic depth.

We found that 29.6% of geolocator positions were recorded over land and 35.6% over waters shallower than 1000 meters (Fig 4). Although the spatial accuracy for geolocator data is low (186 ± 114 km [25], and inland trips are highly unlikely this result indicates that fluttering shearwaters forage very close to land over shallow waters, but occasionally also over pelagic shelf waters around 2000 meter in depth (Fig 4). Average foraging depth was 946 m where migrating birds tend to forage over significantly deeper waters (average 1157 m) than resident birds (738 m; t-test: t_4949_ = 10.416, p<0.00; Fig 4).

### Activity patterns

**Fig 5:**
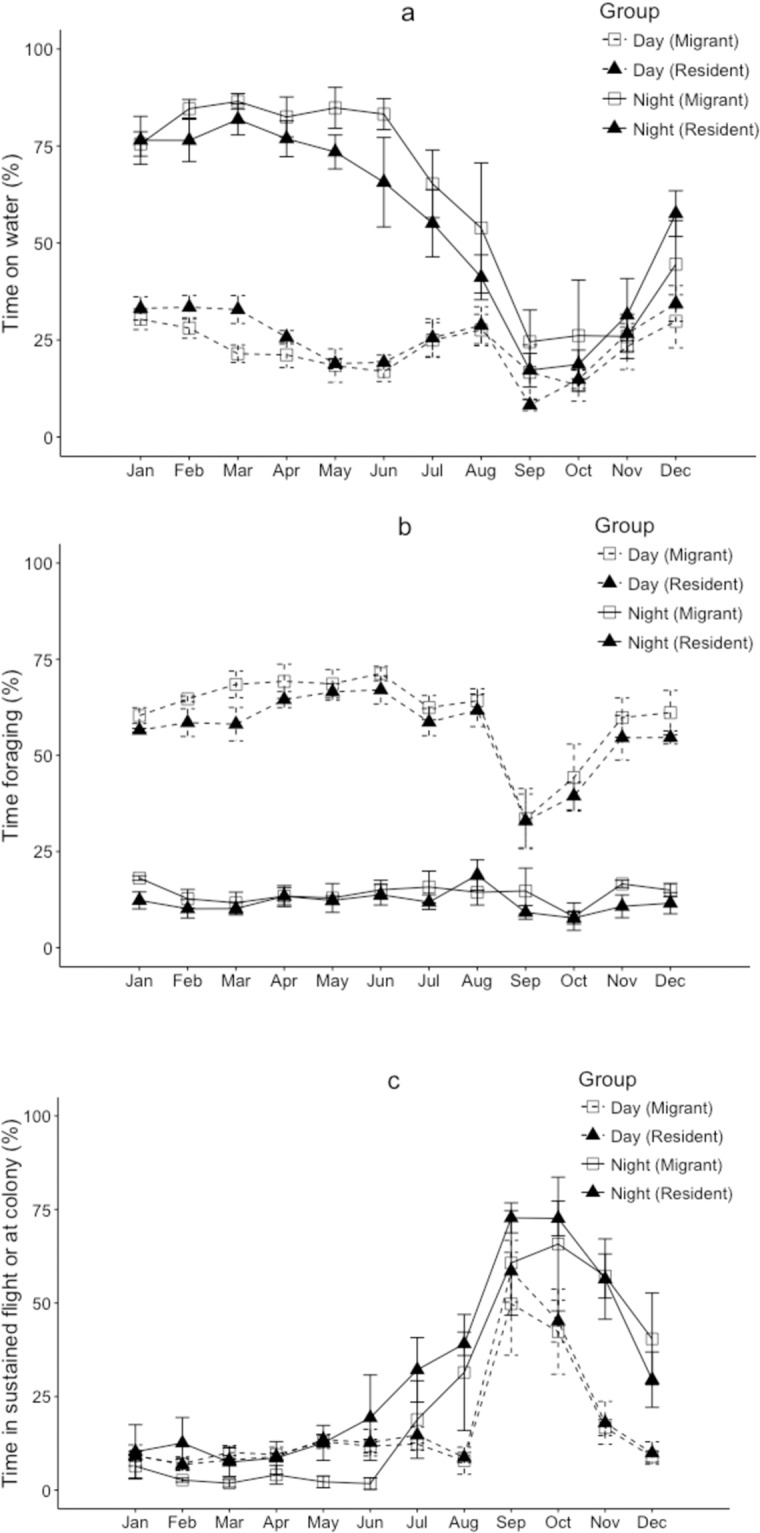
Mean monthly activity metrics during daylight (dotted lines) and nighttime (solid lines) for seven fluttering shearwaters tracked with geolocator immersion loggers from Burgess Island. Open squares represent three birds that migrated to the coast of eastern Australia and closed triangles presents four birds that stayed confined in New Zealand waters year-round.

We found that fluttering shearwaters frequently visited the colonies at night throughout the year (Fig 3). Three birds, which undertook migrations to Australia, stopped visiting their burrows between one and three months before crossing the Tasman Sea. Night-time visits commenced immediately following pre-breeding migration arrival to the Hauraki Gulf in August (Fig 3). Fluttering shearwaters spent a significantly greater proportion of time foraging during daylight hours (61 ± 8%) than in darkness (13.7 ± 5%) (F_(1.47)_ = 5.479; p = 0.0235). We found no significant difference in foraging behaviour between migrating and resident birds (Fig 5, Table 1).

**Table 1:** Activity patterns in % of daylight hours (day) and dark hours (night) (observed mean ± SD). Test statistics (t_df_ and p-values) given for resident (n = 5) and migrating (n = 3) individuals in April, July and December, respectively.

	Flying or on land		Foraging		Time spent on sea surface	
	Day	Night	Day	Night	Day	Night
**April**						
Resident	10.93 ± 2.94	10.85 ± 8.28	67.70 ± 5.93	12.79 ± 3.14	22.37 ± 4.08	75.23 ± 8.59
Migrant	11.22 ± 3.48	3.13 ± 3.36	68.92 ± 6.37	13.15 ± 5.09	19.78 ± 6.08	83.65 ± 8.18
t_1.4_	0.043	0.017	1.22	0.124	0.302	0.424
p	0.846	0.903	0.332	0.741	0.612	0.550
**July**						
Resident	11.80 ± 4.47	35.28 ± 14.27	65.90 ± 3.69	14.56 ± 6.26	27.12 ± 8.48	49.45 ± 16.46
Migrant	10.08 ± 5.00	18.23 ± 24.56	63.31 ± 4.95	15.08 ± 5.88	26.26 ± 6.75	59.56 ± 21.63
t_1.4_	0.418	0.758	0.494	0.67	0.981	0.67
p	0.553	0.304	0.521	0.459	0.378	0.459
**December**						
Resident	14.32 ± 8.53	40.34 ± 18.32	61.54 ± 5.43	11.46 ± 4.95	29.55 ± 7.93	51.83 ±18.69
Migrant	11.80 ± 5.64	46.40 ± 22.73	59.42 ± 11.55	16.75 ± 1.49	28.55 ± 13.98	36.56 ± 22.54
t_1.4_	1.476	2.757	1.332	0.024	1.08	2.101
p	0.291	0.189	0.349	0.884	0.357	0.221

## Discussion

### At-sea distribution

Five of our eight tracked fluttering shearwaters remained remarkably restricted to inshore waters close to the breeding colonies on Burgess Island and Long Island year-round. These findings are supported by at-sea sightings of this species along the coast of north eastern New Zealand and the Cook Strait [41]. The fluttering shearwater is one of only a few mainly non-migratory *Puffinus* species [42]. The oceanic features of the Hauraki Gulf region and the Cook Strait are influenced by a high primary production enhanced by nearshore upwelling [43], and it is possible that the outstanding oceanographic features in these areas allow the fluttering shearwaters to successfully forage in New Zealand waters year-round. Why fluttering shearwaters stay confined to these waters year-round remains to be investigated. A potential explanation indicated by our tracking and bathymetry data (Figs 1 and 4) as well as by at-sea observations is that the fluttering shearwater, in contrast to most other Procellariforms species, forages over a wide range of habitats from inshore waters close to and within harbours and river mouths to pelagic waters far from land ([15, 22, 42], Authors’ pers. obs.). Possibly this “plastic” foraging behaviour allows this species to access a variable, but predictable, food supply year-round and subsequently is able to adapt to seasonal variations [44]. Further studies on seasonal variations in fluttering shearwater diet and trophic niche are required to investigate this hypothesis.

### Migration strategies

Three of the seven individuals breeding on Burgess Island migrated over the Tasman Sea to forage along the coast of eastern Australia (Fig 1). The movements of fluttering shearwaters along the coast of Australia are not well understood, perhaps partly due to their similarity with the closely related Hutton’s shearwater (*Puffinus huttoni*) that also breeds in New Zealand and migrates to Australian waters during the non-breeding season ([41, 45], Haas pers. comm.).

The single fluttering shearwater that was tracked for three years between 2012 and 2015 only migrated in 2012, the other two birds migrated in 2014, suggesting that this is a year dependent effect, rather than a genetic trait, or a movement pattern related to the sex of the bird (e.g. [32]). We found no significant difference in the foraging intensity between migratory and resident individuals, which, in this study, contradicts the hypothesis that migration strategies are related to foraging success and individual fitness [46]. Another possible hypothesis is that the migration strategies of fluttering shearwaters are linked to the breeding success during the previous season. Catry et al. [47] found that failed breeders of Cory’s shearwaters (*Calonectris diomedea*) were more likely to remain in local waters year-round, while all successful breeders migrated to the South Atlantic during the non-breeding season. It remains to be investigated if the migration strategies of the fluttering shearwater correspond to its breeding success in the previous year.

We found birds departed from New Zealand waters on 14 April, 1 July and 2 July, and returned on 12 August, 14 September and 22 August, respectively. We were able to characterise the timing of these migrations quite precisely because it involved direct longitudinal movements, bypassing the usual latitudinal inaccuracy due to equinoxes [25, 48]. At-sea sightings support that numbers of fluttering shearwaters build up in northern New South Wales and southern Queensland in August and September [41], which is surprisingly late because egg-laying peaks around mid-September for fluttering shearwaters breeding in northern New Zealand [18].

This suggests that a significant proportion of the fluttering shearwaters foraging along the coast of Australia during late winter and early spring are juvenile, immature or adult birds taking a sabbatical year from breeding activities. These hypotheses are supported both by ring recoveries of juvenile birds found along the Australian coast [45], and by our data showing that adult fluttering shearwaters departing from Australia to New Zealand in mid-September probably refrained from breeding that year (Fig 3). It is possible, however, that some of the reported sightings of fluttering shearwaters at sea involve the similar looking Hutton’s shearwater.

### Foraging movements in relation to prey

Fluttering shearwaters frequently forage in association with fish shoals together with other seabirds, such as Australasian gannets (*Morus serrator*), fairy prions (*Pachyptila turtur*) and Buller’s shearwaters (*Ardenna bulleri*) (Authors’ pers. obs.). The diet of fluttering shearwaters is poorly studied, but likely consists of small pelagic planktivorous fish, such as pilchard (*Sardinia neopilchardus*), sprat (*Clupea antipodum*) and anchovy (*Engraulis australis*), as well as euphausid shrimp caught in inshore waters during daylight hours (Authors’ pers. obs.). Pilchard and anchovy spawn in the Hauraki Gulf and the Marlborough Sounds in New Zealand in mid to late spring in water temperatures of 16°C to 19°C [49, 50] and coincides with fluttering shearwaters’ chick-rearing in November-December [18]. Spawning runs of pilchards along the Australian south coast, Tasmania and the Bass Strait occurs from mid-spring in October to late-spring in November [49, 50], and along the coast of Southern Queensland in winter and early spring [51]. Spawning biomass of pilchard is largest along northern New South Wales and southern Queensland in August and September [51, 52], which coincides with period when the numbers of fluttering shearwaters are building up along the eastern coast of Australia [52, 53, 54]. These observations support the theory that pilchards are an important food supply and that spawning runs of pilchards influence fluttering shearwaters’ at-sea distribution and migration phenology.

### Implications for conservation management

We found fluttering shearwaters to forage over shallow waters close to the coast (Fig 4). These areas are also intensively used by fisheries [54], which make the fluttering shearwater vulnerable to fisheries interactions and water pollution. More than 166 birds were reported caught in a single gillnet at Whangaparaoa Peninsula in the Hauraki Gulf in May 2009, and bycatch incidents continue to remain underreported [55, 56, 57]. Oil spills may also have a profound impact on fluttering shearwaters [56], and at least 240 individuals were killed by the oil spill from the stranded container ship Rena in the Bay of Plenty north of the Hauraki Gulf in northeastern New Zealand [55, 57]. Further studies based on finer-scale data are required to fully understand the spatial overlap between birds’ inshore foraging distribution and the use of gillnets.

The interaction between small pelagic planktivorous fish and seabirds remains poorly understood in New Zealand waters. Although pilchard fisheries have increased considerably since 1991, especially along the southern coast Australia, the fisheries at present have likely had a negligible impact on marine top predators, such as pelagic seabirds [58]. It will, however, be critical to ensure that future development in fisheries management remains ecologically sustainable for pelagic seabirds in this region [19], including fluttering shearwaters.

### Conclusions

The present study shows that fluttering shearwaters forage in coastal and productive waters of New Zealand year-round and frequently visit their breeding colonies throughout the year. All tracked birds predominately foraged during daylight hours over different habitats from oceanic waters beyond the continental shelf to shallow waters close to the shore. Three out of eight tracked individuals crossed the Tasman Sea to forage along the coast of eastern Tasmanian and Australia during the non-breeding season. These movements overlapped spatially and temporally with the spawning runs of pilchard. Threats at sea remain poorly understood and require further study, but the species is likely vulnerable to gillnet fisheries in inshore waters. Although additional studies of inter-colony variation in different life stages will be important to fully understand the migration strategies and life history of fluttering shearwaters, our study is the first to characterise the year-round foraging behaviour and at-sea distribution of one of New Zealand’s least studied bird species. We hope this new information will help towards a better conservation management of this species as well as to support future ecological studies.

## Supporting information

S1 File Activity budgets for fluttering shearwaters from Burgess Island (*n* = 9). (CSV)

S2 File GLS-locations for fluttering shearwaters from Burgess Island and Long Island (n = 8). (CSV)
